# Cytokine-STAT3 Signaling Axis in Clear Cell Renal Cell Carcinoma: Implications for Tumor Microenvironment and Biomarker Discovery

**DOI:** 10.3390/cancers18121972

**Published:** 2026-06-17

**Authors:** Martina Šutovská, Matúš Dohál, Eduard Gondáš, Jozef Mažerik, Ján Švihra, Lucia Cipková, Soňa Fraňová, Ján Ľupták

**Affiliations:** 1 Department of Pharmacology, Jessenius Faculty of Medicine, Comenius University in Bratislava, Malá Hora 10701/4A, 03601 Martin, Slovakia; matus.dohal@uniba.sk (M.D.); eduard.gondas@uniba.sk (E.G.); mazerik1@uniba.sk (J.M.); cipkova14@uniba.sk (L.C.); sona.franova@uniba.sk (S.F.); 2 Biomedical Center BioMed Martin, Jessenius Faculty of Medicine, Comenius University in Bratislava, Malá Hora 10701/4A, 03601 Martin, Slovakia; 3 Urology Clinic, Jessenius Faculty of Medicine, Comenius University in Bratislava, University Hospital Martin, Kollárova 2, 03659 Martin, Slovakia; jan.svihra2@uniba.sk (J.Š.J.); jan.luptak@uniba.sk (J.Ľ.)

**Keywords:** clear cell renal cell carcinoma, cytokines, STAT3 signaling, tumor microenvironment, urinary biomarkers, inflammation, translational oncology

## Abstract

**Background/Objectives**: Clear cell renal cell carcinoma (ccRCC) is the most prevalent and biologically aggressive subtype of renal cell carcinoma, characterized by pronounced immunogenicity and extensive remodeling of the tumor microenvironment. Chronic inflammation and dysregulated cytokine signaling contribute substantially to tumor progression. Signal transducer and activator of transcription 3 (STAT3) represents a central molecular hub integrating cytokine- and hypoxia-driven pathways. This review aims to summarize current evidence on the cytokine–STAT3 signaling axis in ccRCC and to evaluate its translational relevance for biomarker development. **Methods**: A narrative review of the literature was conducted using PubMed, Scopus, and Web of Science databases. Experimental, translational, and clinical studies addressing cytokine signaling, STAT3 activation, tumor microenvironment interactions, and biomarker development in ccRCC were evaluated. Particular attention was given to studies analyzing cytokine profiles in tumor tissue, plasma, and urine, as well as their associations with STAT3 activation and clinicopathological parameters. **Results**: Accumulating evidence indicates that ccRCC exhibits a complex, compartment-specific cytokine signature involving interleukins, chemokines, and tumor necrosis factor (TNF)-related cytokines. Among these mediators, IL-6, IL-8, and selected chemokines such as CXCL10 appear particularly relevant due to their associations with tumor progression, immune modulation, and clinical outcome. Many of these mediators converge on persistent STAT3 activation, which promotes tumor cell survival, angiogenesis, immune suppression, and metastatic potential. Tissue-based analyses demonstrate correlations between altered cytokine expression and STAT3 activation, while urinary cytokine profiles reflect tumor-associated inflammatory processes in a non-invasive manner. Plasma cytokines appear to capture broader systemic inflammatory responses. **Conclusions**: The cytokine–STAT3 axis represents a biologically plausible signaling network associated with tumor progression and immune modulation in ccRCC. By integrating evidence from cytokine profiling in tumor tissue, plasma, and urine with current knowledge of STAT3 signaling, this review highlights the importance of compartment-specific inflammatory signatures in understanding ccRCC biology and their potential relevance for biomarker discovery. Integrative approaches combining cytokine profiling with functional assessment of STAT3 activation may improve disease characterization and support the development of diagnostic and prognostic biomarkers, although rigorous clinical validation remains necessary.

## Simple Summary

Clear cell renal cell carcinoma (ccRCC) is characterized by pronounced immunogenicity and extensive remodeling of the tumor microenvironment, driven by chronic inflammation and dysregulated cytokine signaling. A central mediator of these processes is signal transducer and activator of transcription 3 (STAT3), which integrates cytokine- and hypoxia-induced pathways and promotes tumor cell survival, angiogenesis, immune suppression, and metastatic progression. This review summarizes current knowledge on cytokine networks in ccRCC and their functional convergence on STAT3 signaling. Particular attention is given to cytokine profiling across tumor tissue, plasma, and urine, highlighting the compartment-specific nature of inflammatory signaling. While tissue-based analyses provide mechanistic insight, urinary cytokines represent a promising non-invasive biomarker source reflecting tumor-associated inflammation. The integration of cytokine patterns with STAT3 activation status may enhance diagnostic and prognostic precision. Although clinical implementation requires further validation, the cytokine–STAT3 axis represents a biologically relevant and translationally promising target in ccRCC.

## 1. Introduction

Clear cell renal cell carcinoma (ccRCC) represents the most prevalent and biologically aggressive histological subtype of renal cell carcinoma (RCC), accounting for approximately 70–80% of all renal malignancies [1,2]. Despite substantial progress in imaging techniques and surgical management, ccRCC remains a major clinical challenge due to its largely asymptomatic course in early stages and its pronounced tendency toward progression and metastasis. A significant proportion of patients are diagnosed incidentally, often at an advanced stage, when curative treatment options are limited and long-term survival remains poor [3]. While localized disease can frequently be managed successfully by surgical resection, metastatic ccRCC continues to be associated with unfavorable outcomes despite the introduction of targeted therapies and immune checkpoint inhibitors [4,5].

One of the defining biological features of ccRCC is its intrinsic immunogenicity and the profound remodeling of the tumor microenvironment (TME). Unlike many other solid tumors, ccRCC is characterized by extensive immune cell infiltration, high vascular density, and marked alterations in inflammatory signaling pathways [6,7]. These characteristics reflect, at least in part, the molecular consequences of von Hippel–Lindau (VHL) tumor suppressor gene inactivation [8] and subsequent dysregulation of hypoxia-inducible signaling. However, the immunobiological landscape of ccRCC extends well beyond hypoxia-driven angiogenesis and includes complex cytokine networks that actively shape tumor progression, immune escape, and therapeutic responsiveness [9].

Cytokines constitute a heterogeneous group of soluble signaling molecules that orchestrate intercellular communication within the immune system and between tumor cells and their microenvironment. In the context of cancer, cytokines exert pleiotropic and often context-dependent effects, influencing cell proliferation, apoptosis, angiogenesis, immune surveillance, and metastatic dissemination [10,11]. In ccRCC, both pro-inflammatory and anti-inflammatory cytokines have been implicated in disease initiation and progression, as well as in shaping the balance between anti-tumor immunity and immunosuppression. Importantly, many of these cytokines converge on common intracellular signaling pathways, among which the Janus kinase/signal transducer and activator of transcription (JAK/STAT) pathway plays a central role [12,13]. The JAK/STAT pathway comprises several STAT family members (STAT1–STAT6), each with distinct and partially overlapping biological functions.

Among them, STAT3 has emerged as a key molecular hub linking cytokine signaling to oncogenic processes. Persistent activation of STAT3 has been documented across a wide spectrum of malignancies and is associated with enhanced tumor cell survival, proliferation, angiogenesis, and metastatic potential [14,15]. In addition to its direct effects on malignant cells, STAT3 critically regulates the functional phenotype of immune cells within the TME, promoting immunosuppressive mechanisms that facilitate tumor immune evasion. In ccRCC, accumulating evidence suggests that aberrant STAT3 signaling represents an important driver of tumor biology and may serve as a functional integrator of cytokine-mediated signals derived from both tumor and stromal compartments [16,17].

Despite extensive research into individual cytokines and signaling pathways, clinically applicable biomarkers for early detection, risk stratification, and treatment monitoring in ccRCC remain lacking [18]. From a translational perspective, there is a growing interest in identifying non-invasive or minimally invasive biomarkers that reflect tumor-associated inflammatory processes. In this regard, cytokine profiling in various biological matrices, including tumor tissue, blood, and urine, has gained increasing attention [19]. Urine, in particular, represents an attractive source of biomarkers in renal malignancies due to its direct contact with the tumor-bearing organ and its accessibility in routine clinical practice [20].

Despite substantial progress in understanding the immunobiology of ccRCC, current knowledge remains fragmented across several research domains, including tumor microenvironment biology, cytokine signaling, STAT3 activation, and biomarker development. Previous reviews have primarily focused on individual aspects of ccRCC pathogenesis, immune regulation, or therapeutic strategies, whereas the integration of cytokine-mediated signaling with STAT3 activation across different biological compartments has been addressed only to a limited extent. In particular, the relationship between tissue, plasma, and urinary cytokine profiles and their potential contribution to biomarker discovery remains insufficiently synthesized. Addressing this knowledge gap is especially relevant in the era of precision oncology, where the identification of clinically useful, minimally invasive biomarkers and biologically informed therapeutic targets has become a major research priority.

The present review aims to provide a comprehensive overview of the cytokine–STAT3 signaling axis in ccRCC, with a particular focus on its role within the TME and its potential relevance for biomarker discovery. By integrating current literature describing cytokine profiling in tumor tissue, plasma, and urine samples of ccRCC patients, this article seeks to highlight emerging concepts in ccRCC immunobiology and to discuss their translational implications. Understanding the complex interplay between cytokines and STAT3 signaling may ultimately contribute to the development of novel diagnostic strategies and therapeutic approaches tailored to the immunobiochemical landscape of ccRCC.

## 2. Material and Methods

### Literature Search Strategy

This article was prepared as a narrative review of the current literature addressing cytokine signaling, STAT3 activation, tumor microenvironment interactions, and biomarker development in ccRCC. Relevant publications were identified through searches of PubMed, Scopus, and Web of Science databases up to January 2026. Search terms included combinations of the following keywords: “clear cell renal cell carcinoma”, “renal cell carcinoma”, “cytokines”, “interleukins”, “chemokines”, “STAT3”, “JAK/STAT signaling”, “tumor microenvironment”, “urinary biomarkers”, “plasma biomarkers”, “inflammation”, and “immune regulation”.

Priority was given to original research articles, translational studies, systematic reviews, and meta-analyses published in peer-reviewed journals. Additional relevant publications were identified through manual screening of reference lists.

Studies were selected based on their relevance to cytokine-mediated signaling pathways, STAT3 activation, tumor microenvironment biology, and biomarker research in ccRCC. Experimental studies, clinical investigations, and translational studies were considered. Publications not directly related to ccRCC, studies lacking sufficient methodological information, and non-English articles were generally excluded.

As this work was designed as a narrative review, no formal systematic-review methodology, risk-of-bias assessment, or quantitative meta-analysis was performed. The objective was to provide a comprehensive overview of current knowledge and to identify emerging concepts relevant to cytokine–STAT3 signaling in ccRCC.

## 3. Clear Cell Renal Cell Carcinoma and the Tumor Microenvironment

**Figure 1:**
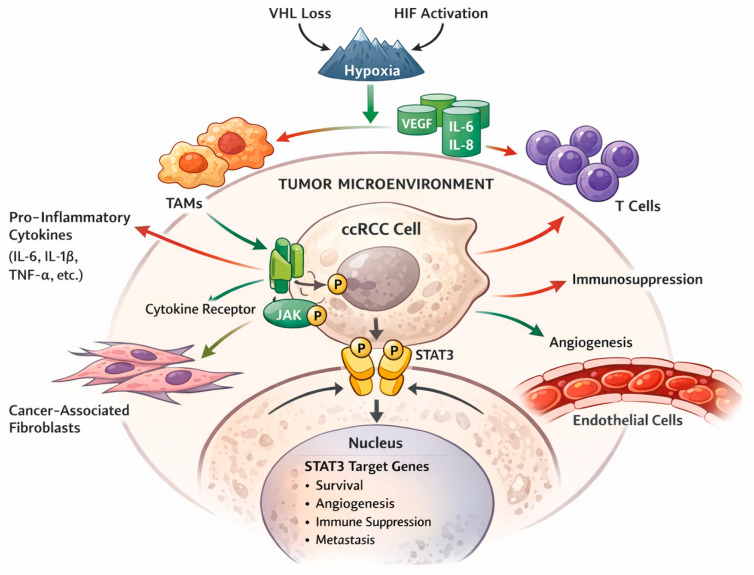
Cytokine–STAT3 signaling within the tumor microenvironment (TME) of clear cell renal cell carcinoma (ccRCC). Loss of von Hippel–Lindau (VHL) function leads to constitutive activation of hypoxia-inducible factors (HIFs), resulting in enhanced production of pro-angiogenic and pro-inflammatory mediators, including vascular endothelial growth factor (VEGF) and cytokines such as interleukin-6 (IL-6) and interleukin-8 (IL-8). These cytokines are produced by tumor cells as well as by components of the TME, including tumor-associated macrophages (TAMs), T lymphocytes (T Cells), cancer-associated fibroblasts, and endothelial cells. Cytokine binding to their cognate receptors activates the Janus kinase (JAK)/signal transducer and activator of transcription 3 (STAT3) pathway in ccRCC cells, leading to STAT3 phosphorylation, dimerization, and nuclear translocation. Activated STAT3 regulates the transcription of target genes involved in tumor cell survival, angiogenesis, immune suppression, and metastatic potential, thereby reinforcing a tumor-promoting microenvironment. The image was generated with the assistance of an artificial intelligence tool (ChatGPT, OpenAI) based on author-defined input and was subsequently reviewed and refined by the authors to ensure scientific accuracy.

The TME of ccRCC is a highly dynamic and heterogeneous ecosystem composed of malignant epithelial cells, immune cells, stromal elements, endothelial cells, and a complex extracellular matrix [21]. This microenvironment is not merely a passive scaffold for tumor growth but actively participates in tumor initiation, progression, and therapeutic resistance. In ccRCC (Figure 1), the TME is profoundly shaped by hypoxia-driven molecular alterations, chronic inflammation, and sustained immune modulation, resulting in a unique immunobiological profile that distinguishes this tumor entity from many other solid malignancies [6,22].

A hallmark of ccRCC is the constitutive activation of hypoxia-responsive pathways, primarily resulting from inactivation of the VHL tumor suppressor gene [23]. Loss of VHL function leads to stabilization of hypoxia-inducible factors even under normoxic conditions, driving transcriptional programs that promote angiogenesis, metabolic reprogramming, and cell survival [24]. While the vascular phenotype of ccRCC has been extensively studied, hypoxia-induced signaling also exerts profound effects on immune regulation and cytokine production within the TME. Hypoxic conditions favor the expression of multiple cytokines and chemokines that recruit immune cells, modulate their functional state, and contribute to the establishment of an immunosuppressive milieu [25].

Immune cell infiltration represents another defining feature of the ccRCC microenvironment. Tumor-infiltrating lymphocytes, macrophages, dendritic cells, natural killer cells, and myeloid-derived suppressor cells are commonly detected within ccRCC lesions [26]. However, the mere presence of immune cells does not necessarily translate into effective anti-tumor immunity. On the contrary, numerous studies have demonstrated that ccRCC is often associated with dysfunctional or exhausted immune phenotypes, characterized by impaired cytotoxic activity and the dominance of immunoregulatory cell populations [27]. Cytokines play a pivotal role in orchestrating these immune dynamics, influencing both the recruitment and functional polarization of immune cells [10].

Tumor-associated macrophages (TAMs) are among the most abundant immune cells in ccRCC and represent a major source of cytokines within the TME [28]. Depending on the local cytokine milieu, macrophages may adopt pro-inflammatory or anti-inflammatory phenotypes, each exerting distinct effects on tumor biology. In ccRCC, TAMs frequently exhibit features associated with immunosuppression, angiogenesis, and tissue remodeling [29]. The cytokines produced by these cells contribute to sustained STAT3 activation in both immune and tumor cells, thereby reinforcing a tumor-promoting feedback loop [30].

Beyond immune cells, stromal fibroblasts and endothelial cells also actively contribute to cytokine signaling in ccRCC. Cancer-associated fibroblasts secrete a variety of cytokines and growth factors that support tumor growth and modulate immune cell behavior, while endothelial cells participate in inflammatory signaling linked to angiogenesis and leukocyte trafficking [31,32]. The interplay between these cellular compartments underscores the complexity of cytokine networks operating within the ccRCC microenvironment.

Importantly, the cytokine landscape of the TME is not static but evolves during disease progression. Early-stage tumors may exhibit cytokine profiles distinct from those observed in advanced or metastatic disease, reflecting shifts in immune surveillance, tumor cell adaptation, and stromal remodeling [33]. These dynamic changes have significant implications for disease prognosis and therapeutic response, particularly in the context of immunomodulatory treatments. Consequently, a detailed understanding of cytokine-driven processes within the ccRCC microenvironment is essential for identifying clinically relevant biomarkers and for optimizing therapeutic strategies targeting immune-related pathways [34,35].

The cellular and non-cellular components of the TME contribute to the establishment of a cytokine-rich milieu that supports persistent inflammatory signaling. Cytokines produced by tumor cells, tumor-associated macrophages, cancer-associated fibroblasts, and infiltrating immune cells may activate STAT3 directly or indirectly, thereby promoting tumor cell survival, angiogenesis, immune evasion, and metastatic progression. Among the most relevant mediators, IL-6 has emerged as a major activator of the JAK/STAT3 pathway, while other cytokines and chemokines contribute to shaping the inflammatory network within the TME. These observations suggest that the biological significance of the TME extends beyond its cellular composition and includes dynamic cytokine-driven signaling processes that converge on STAT3 activation.

## 4. Cytokines in Clear Cell Renal Cell Carcinoma Pathophysiology

Cytokines (Table 1) represent central regulators of immune and inflammatory responses and play a multifaceted role in the pathophysiology of ccRCC. Within the TME, cytokines act as key mediators of communication between malignant cells and surrounding stromal and immune compartments, thereby influencing tumor growth, immune surveillance, angiogenesis, and metastatic dissemination [36,37].

In ccRCC, the cytokine milieu is shaped by hypoxia-driven signaling, genetic alterations intrinsic to tumor cells, and chronic inflammatory stimuli, resulting in a complex network of soluble factors with both tumor-promoting and tumor-suppressive properties [44].

Unlike classical inflammation-driven malignancies, ccRCC exhibits a distinctive cytokine profile that reflects its unique molecular background. Constitutive activation of hypoxia-inducible pathways following VHL inactivation promotes the expression of multiple cytokines and growth factors that support angiogenesis and immune modulation. At the same time, infiltrating immune cells contribute additional layers of cytokine signaling, creating a highly dynamic and context-dependent cytokine landscape. Importantly, the biological effects of individual cytokines cannot be considered in isolation, as their actions are tightly interconnected through shared signaling pathways and feedback mechanisms [6,45].

### 4.1. Interleukins

Interleukins constitute one of the most extensively studied cytokine families in ccRCC and have been implicated in diverse aspects of tumor biology. Several interleukins exhibit altered expression in ccRCC compared to non-malignant renal tissue, underscoring their potential relevance for disease progression and clinical outcome. Among these, interleukin (IL)-6 has attracted particular attention due to its strong association with systemic inflammation, tumor aggressiveness, and poor prognosis in RCC patients [44].

IL-6 is produced by tumor cells, tumor-associated macrophages, and other stromal components within the ccRCC microenvironment. Through activation of the JAK/STAT3 signaling cascade, IL-6 promotes tumor cell proliferation, resistance to apoptosis, and metabolic adaptation. Elevated circulating IL-6 levels have been repeatedly linked to advanced disease stage and unfavorable survival outcomes, highlighting its potential utility as a prognostic biomarker. Beyond its direct effects on tumor cells, IL-6 also contributes to immune dysregulation by impairing effective anti-tumor immune responses and fostering immunosuppressive cell populations [14,46].

Other interleukins, including IL-1β, IL-8, IL-10, and IL-17, have also been implicated in ccRCC pathophysiology [39]. IL-1β acts as a potent pro-inflammatory mediator that enhances tumor invasiveness and promotes angiogenesis through induction of downstream cytokines and growth factors [47,48]. IL-8, a chemokine with strong pro-angiogenic properties, has been associated with tumor progression and resistance to targeted therapies in RCC [49,50]. In contrast, IL-10 exerts predominantly immunosuppressive effects by inhibiting antigen presentation and cytotoxic T-cell activity, thereby facilitating immune escape [51]. IL-17 has been reported to contribute to chronic inflammation and angiogenesis, although its precise role in ccRCC remains incompletely defined and may depend on the cellular context and disease stage [52]. Available evidence regarding IL-17 in ccRCC remains limited and partially inconsistent. While some studies suggest a pro-tumorigenic role through promotion of angiogenesis and inflammatory signaling, the precise contribution of IL-17-producing immune-cell subsets and their relationship to disease progression have not yet been fully elucidated. Further mechanistic studies are therefore required to clarify the biological significance of IL-17 signaling in ccRCC.

Collectively, these findings illustrate the dualistic nature of interleukin signaling in ccRCC, where pro-inflammatory and anti-inflammatory interleukins coexist and jointly shape tumor behavior. The balance between these opposing signals appears to be critical for determining disease trajectory and therapeutic responsiveness.

### 4.2. Chemokines and Chemokine Receptors

Chemokines represent another essential component of the cytokine network in ccRCC, primarily mediating the recruitment and spatial organization of immune cells within the TME. Members of the CC and CXC chemokine families are frequently dysregulated in ccRCC and contribute to both immune cell infiltration and angiogenesis [53,54].

Pro-angiogenic CXC chemokines play a prominent role in promoting neovascularization in ccRCC [55]. By stimulating endothelial cell proliferation and migration, these chemokines support the highly vascularized phenotype characteristic of this tumor type. At the same time, chemokines such as CXCL8 and CXCL12 facilitate the recruitment of immunosuppressive myeloid cells and regulatory T cells, thereby reinforcing an immune-tolerant microenvironment [56,57].

Chemokine receptors expressed on tumor cells and immune cells further modulate these effects by enabling autocrine and paracrine signaling loops [58]. Aberrant chemokine receptor expression has been linked to enhanced tumor cell motility, invasion, and metastatic potential [59]. Importantly, several chemokines and their receptors have been associated with disease stage and clinical outcome in ccRCC, underscoring their potential relevance as biomarkers and therapeutic targets [6,40].

### 4.3. Tumor Necrosis Factor and Related Cytokines

Tumor necrosis factor alpha (TNF-α) represents a prototypical pro-inflammatory cytokine with context-dependent effects in cancer. In ccRCC, TNF-α is produced by immune cells within the TME and contributes to chronic inflammation and immune modulation. While acute TNF-α signaling may exert anti-tumor effects, sustained exposure promotes tumor progression by inducing survival pathways, angiogenesis, and immune suppression [60,61].

TNF-α signaling intersects with multiple intracellular pathways, including nuclear factor kappa B and JAK/STAT, thereby amplifying inflammatory cascades within the TME [62]. Through these mechanisms, TNF-α contributes to the maintenance of a cytokine-rich milieu that favors tumor persistence and progression. Other members of the TNF superfamily similarly participate in shaping immune responses and may influence ccRCC biology through overlapping signaling networks [6,63].

### 4.4. Integrated Cytokine Networks in ccRCC

Rather than acting as isolated mediators, cytokines in ccRCC form highly interconnected signaling networks that collectively determine the functional state of the TME. Crosstalk between interleukins, chemokines, and TNF-related cytokines enables fine-tuned regulation of immune cell behavior, angiogenesis, and tumor cell survival [6,10]. Central signaling nodes such as STAT3 integrate these diverse inputs and translate them into coordinated transcriptional programs that support tumor progression [14,64].

Importantly, alterations in cytokine profiles have been observed not only within tumor tissue but also in systemic circulation and urine of ccRCC patients, suggesting that local inflammatory processes are reflected at the organismal level. These observations provide a strong rationale for exploring cytokine signatures as potential non-invasive biomarkers and for investigating their relationship with key intracellular signaling pathways such as STAT3 [65,66]. The following sections will therefore focus on STAT3 signaling in ccRCC and its functional interplay with cytokine-mediated processes.

## 5. Molecular Mechanisms of STAT3 Activation and Regulation

STAT3 is a latent cytoplasmic transcription factor that occupies a central position in cytokine-mediated signaling and has emerged as a key driver of tumor-promoting processes in a wide range of malignancies, including ccRCC [14,64]. In physiological conditions, STAT3 activation is transient and tightly regulated, enabling controlled cellular responses to extracellular stimuli. In contrast, persistent or aberrant STAT3 activation is a hallmark of many cancers and contributes to malignant transformation, tumor progression, and immune evasion [64,67].

### 5.1. Canonical STAT3 Activation Pathway

**Figure 2:**
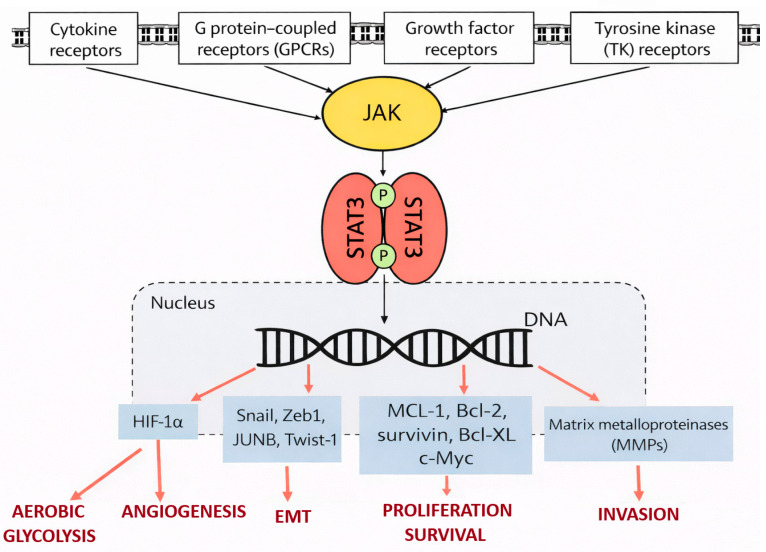
Canonical JAK/STAT3 signaling pathway and selected downstream transcriptional targets relevant to cancer biology. Schematic representation of STAT3 activation through the canonical cytokine receptor–JAK signaling pathway and its major downstream transcriptional effects. Binding of cytokines to their cognate receptors induces activation of Janus kinases (JAKs), leading to phosphorylation of STAT3, STAT3 dimerization, and subsequent nuclear translocation. In the nucleus, activated STAT3 binds to specific DNA response elements and regulates the transcription of genes involved in multiple cancer-related processes. STAT3-dependent transcription contributes to metabolic reprogramming through regulation of hypoxia-inducible factor-1α (HIF-1α), promotes angiogenesis, supports epithelial–mesenchymal transition (EMT) through transcription factors such as Snail, ZEB1, JUNB, and TWIST1, enhances cell proliferation and survival via induction of MCL-1, BCL-2, survivin, BCL-XL, and c-MYC, and facilitates tumor invasion through increased expression of matrix metalloproteinases (MMPs). Although STAT3 may also be activated downstream of growth factor receptors and other signaling pathways, these mechanisms are not depicted in detail, and the schematic is intended to illustrate the canonical cytokine receptor–JAK–STAT3 signaling route. The image was generated with the assistance of an artificial intelligence tool (ChatGPT, OpenAI) based on author-defined input and was subsequently reviewed and refined by the authors to ensure scientific accuracy.

STAT3 is primarily activated through phosphorylation of a critical tyrosine residue, which is mediated by upstream kinases following engagement of cytokine or growth factor receptors. Cytokines such as interleukins and members of the interferon family represent major physiological activators of STAT3 signaling. Upon receptor engagement, associated Janus kinases catalyze STAT3 phosphorylation, leading to its dimerization, nuclear translocation, and binding to specific DNA response elements [64,67]. This process initiates the transcription of a broad array of target genes involved in cell survival, proliferation, metabolism, and immune regulation (Figure 2).

The intensity and duration of STAT3 signaling are controlled by multiple negative regulatory mechanisms, including suppressors of cytokine signaling, protein inhibitors of activated STAT, and protein tyrosine phosphatases [68]. Disruption of these regulatory checkpoints can result in sustained STAT3 activation, thereby amplifying oncogenic signaling cascades [14,15]. In ccRCC, dysregulation of STAT3 signaling may arise from excessive cytokine stimulation, altered expression of regulatory proteins, or crosstalk with hypoxia-responsive pathways [25].

### 5.2. Oncogenic Functions of STAT3 in ccRCC Cells

In tumor cells, STAT3 activation promotes multiple hallmarks of cancer. One of its most prominent functions is the enhancement of tumor cell survival through upregulation of anti-apoptotic proteins and cell cycle regulators. By inducing transcriptional programs that favor proliferation and resistance to cell death, STAT3 contributes to tumor growth and therapy resistance [64,68,69]. In ccRCC, where tumor cells are frequently exposed to hypoxic and inflammatory stress, STAT3-mediated survival signaling may represent a critical adaptive mechanism.

STAT3 also plays a pivotal role in angiogenesis, a defining feature of ccRCC. Through transcriptional regulation of pro-angiogenic factors, STAT3 cooperates with hypoxia-inducible signaling to sustain the highly vascularized phenotype of these tumors [25,70]. This cooperative interaction underscores the functional convergence of hypoxia-driven and cytokine-driven pathways in ccRCC pathobiology.

In addition, STAT3 has been implicated in epithelial–mesenchymal transition, tumor cell motility, and metastatic dissemination. By modulating the expression of genes involved in cell adhesion and extracellular matrix remodeling, STAT3 facilitates invasive behavior and may contribute to disease progression toward advanced and metastatic stages [15,71].

### 5.3. STAT3 as a Modulator of the Tumor Immune Microenvironment

Beyond its tumor cell–intrinsic effects, STAT3 exerts profound influence on the immune landscape of ccRCC. STAT3 activation in immune cells within the TME promotes the development of immunosuppressive phenotypes and impairs effective anti-tumor immunity [6,68]. In myeloid cells, STAT3 signaling drives the expansion and functional polarization of tumor-associated macrophages and myeloid-derived suppressor cells, both of which are key contributors to immune evasion [72].

STAT3 also interferes with dendritic cell maturation and antigen presentation, thereby limiting the initiation of robust adaptive immune responses [73]. In T lymphocytes, STAT3 signaling has been associated with reduced cytotoxic activity and increased differentiation toward regulatory T-cell phenotypes [74]. Collectively, these effects create an immunosuppressive microenvironment that favors tumor persistence despite the presence of immune infiltrates.

### 5.4. STAT3 at the Intersection of Cytokine and Hypoxia Signaling in ccRCC

A distinctive feature of ccRCC is the convergence of cytokine-mediated and hypoxia-driven signaling pathways on STAT3. Hypoxic conditions and stabilization of hypoxia-inducible factors enhance the expression of multiple cytokines capable of activating STAT3, while STAT3 itself can modulate hypoxia-responsive gene expression. This bidirectional interaction establishes a self-reinforcing signaling network that supports tumor growth, angiogenesis, and immune modulation [75].

Beyond its effects on angiogenesis, hypoxia may influence cytokine production, immune-cell recruitment, and metabolic adaptation within the TME. Through interactions with hypoxia-inducible pathways, STAT3 contributes to the coordination of inflammatory signaling, immune remodeling, and metabolic reprogramming, thereby linking cytokine-mediated responses with the characteristic hypoxic phenotype of ccRCC. This functional interplay may further promote tumor progression and therapeutic resistance.

The integration of cytokine and hypoxia signals by STAT3 positions this transcription factor as a central molecular hub in ccRCC pathophysiology [76]. Importantly, the degree of STAT3 activation may reflect the cumulative impact of diverse microenvironmental cues, making it a potential indicator of tumor aggressiveness and biological behavior.

### 5.5. Non-Canonical STAT3 Signaling and Crosstalk with Other Pathways

In addition to canonical activation through JAK-mediated phosphorylation, STAT3 participates in complex interactions with several signaling pathways implicated in ccRCC progression. Crosstalk between STAT3 and NF-κB may contribute to the maintenance of chronic inflammatory signaling, as both transcription factors regulate overlapping sets of genes involved in cell survival, cytokine production, and immune modulation [77,78]. Furthermore, interactions between STAT3 and the PI3K/AKT pathway may enhance tumor cell proliferation, resistance to apoptosis, and metabolic adaptation [78,79]. Emerging evidence also suggests a role for STAT3 in the regulation of cellular metabolism through effects on mitochondrial function and metabolic reprogramming [78]. Although these non-canonical mechanisms remain less well characterized in ccRCC than classical cytokine-induced JAK/STAT3 signaling, they further support the concept of STAT3 as a central integrator of multiple oncogenic and inflammatory signals within the TME [77,79].

Emerging evidence also suggests interactions between STAT3 and additional regulatory pathways, including TGF-β signaling and Hippo pathway effectors YAP (Yes-associated protein) and TAZ (transcriptional coactivator with PDZ-binding motif), further highlighting the complexity of signaling networks involved in ccRCC progression and tumor microenvironment remodeling [80].

### 5.6. Implications for Biomarker Development and Therapeutic Targeting

Given its central role in integrating oncogenic and immunomodulatory signals, STAT3 represents an attractive candidate for biomarker development and therapeutic intervention in ccRCC [68]. Assessment of activated STAT3 levels in tumor tissue may provide insights into the inflammatory and immunological status of the TME. Moreover, correlations between STAT3 activation and specific cytokine profiles could enhance the biological interpretability of cytokine-based biomarkers [6,65].

From a therapeutic perspective, targeting STAT3 signaling has the potential to disrupt both tumor cell survival pathways and immunosuppressive mechanisms within the TME. Although direct STAT3 inhibitors have faced clinical challenges, modulation of upstream cytokine signaling or combination strategies with existing immunotherapies may offer promising avenues for future treatment development [81,82].

## 6. Evidence Linking Cytokine Signaling and STAT3 Activation in Clear Cell Renal Cell Carcinoma

The biological effects of cytokines in ccRCC are largely mediated through intracellular signaling pathways that integrate extracellular cues into coordinated transcriptional programs. Among these pathways, STAT3 occupies a pivotal position by functioning as a convergence point for multiple cytokine-driven signals [64,68]. The concept of cytokine–STAT3 crosstalk is therefore central to understanding how inflammatory and immunoregulatory processes shape ccRCC progression, TME remodeling, and clinical behavior [83].

### 6.1. Conceptual Framework of Cytokine–STAT3 Interactions

**Figure 3:**
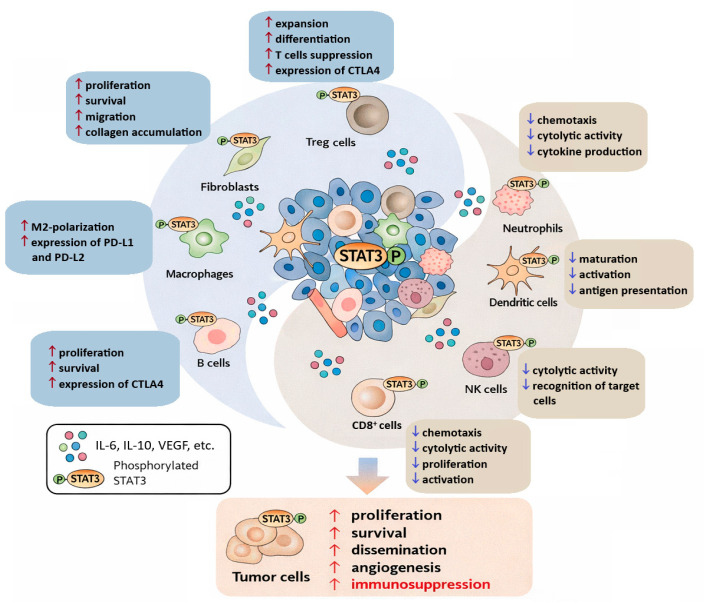
Complex effects of STAT3 (signal transducer and activator of transcription 3) within the tumor microenvironment (TME). Phosphorylation-activated STAT3 (p-STAT3) reinforces the immunosuppressive phenotype of the TME through two complementary mechanisms. On the one hand, p-STAT3 promotes the accumulation and functional activity of pro-tumorigenic immune cell populations, including regulatory T lymphocytes (Tregs), B cells, and M2-polarized macrophages. On the other hand, p-STAT3 suppresses the antitumor functions of neutrophils, CD8^+^ T lymphocytes, dendritic cells, and natural killer (NK) cells. The combined effects of STAT3 activation lead to enhanced tumor progression and dissemination within the host organism. The image was generated with the assistance of an artificial intelligence tool (ChatGPT, OpenAI) based on author-defined input and was subsequently reviewed and refined by the authors to ensure scientific accuracy. IL-interleukin; VEGF—vascular endothelial growth factor; CTLA-4—cytotoxic T-lymphocyte–associated protein 4.

STAT3 represents a central intracellular signaling node integrating cytokine-mediated inflammatory cues within the TME. In ccRCC, STAT3 activation (Figure 3) is not driven by a single dominant cytokine but rather reflects the cumulative input of multiple inflammatory mediators present in the tumor milieu [44]. Cytokines such as IL-6, IL-10, G-CSF, and chemokines including CXCL10 and CCL3 are abundantly expressed within ccRCC tissue and converge on STAT3 signaling through activation of the JAK/STAT pathway [84].

Importantly, STAT3 does not merely act as a downstream effector of cytokine signaling but actively participates in feedback loops that modulate cytokine production. Activated STAT3 can induce transcription of cytokines and cytokine receptors, thereby reinforcing inflammatory signaling and sustaining its own activation [85]. This reciprocal regulation establishes self-perpetuating signaling circuits that promote tumor persistence and progression.

In ccRCC, this signaling architecture is further influenced by hypoxia-driven pathways, metabolic stress, and immune checkpoint regulation. The intersection of these processes amplifies the functional relevance of STAT3 as a molecular hub and underscores its role as an integrator of diverse microenvironmental signals [15,86].

### 6.2. Evidence from Experimental and Clinical Studies

Experimental studies across diverse cancer models have demonstrated that cytokine-mediated STAT3 activation promotes tumor cell survival, angiogenesis, and immune escape [77,79]. In ccRCC, clinical and translational studies consistently point to persistent STAT3 activation as a feature associated with aggressive tumor behavior and immune dysregulation [6,72].

Tissue-based analyses have shown that ccRCC tumors exhibit a distinct inflammatory cytokine signature compared with non-malignant renal parenchyma. Elevated levels of cytokines and chemokines involved in myeloid cell recruitment, T-cell regulation, and angiogenesis have been reported, providing indirect evidence for sustained STAT3 pathway engagement [87,88]. Clinical correlations further suggest that STAT3 activation is linked to advanced disease stage and unfavorable biological characteristics, although the precise contribution of individual cytokines appears to be context-dependent [78,89].

### 6.3. Integration of Tissue-Based Cytokine Profiles with STAT3 Activation

Combined evaluation of tissue-based cytokine profiling with STAT3 signaling analyses suggests potential interactions between inflammatory cytokine networks and STAT3 activation in ccRCC. Profiling studies of ccRCC tumor tissue have demonstrated that multiple inflammatory mediators, including IL-6, IL-10, CXCL10, CCL3, CCL4, and G-CSF, are present at altered levels compared with non-malignant renal tissue [90,91]. Importantly, several of these cytokines show significant associations with STAT3 expression or activation status within tumor tissue [15,84].

Correlation analyses have revealed that STAT3 activation is not uniformly associated with all cytokines but rather selectively linked to specific inflammatory mediators, supporting a multimodal rather than linear activation model [92]. These observations are consistent with the hypothesis that STAT3 may function as an integrative signaling hub linking multiple inflammatory signals within the ccRCC TME [88].

Among the identified mediators, IL-6 represents the best-characterized activator of STAT3 signaling in ccRCC, whereas the functional significance of associations observed for other cytokines, including CXCL10, CCL3, and G-CSF, remains less well established and requires further mechanistic validation.

From a biological perspective, the observed associations between cytokine patterns and STAT3 activation may contribute to the coexistence of chronic inflammation, immune modulation, and tumor progression in ccRCC. The presence of multiple STAT3-associated cytokines further supports the concept that targeting single cytokines may be insufficient to disrupt STAT3-driven tumor biology [93].

### 6.4. Crosstalk Across Biological Compartments: Tissue, Plasma, and Urine

Although STAT3 activation occurs primarily within tumor tissue, cytokine-mediated signaling in ccRCC extends beyond the local microenvironment. Cytokines associated with STAT3 activation in tumor tissue can also be detected in plasma and urine, indicating partial spillover of tumor-associated inflammatory processes into systemic and excretory compartments [94].

Importantly, the strength and specificity of cytokine–STAT3 associations differ across biological matrices. While intratumoral cytokines are most closely linked to STAT3 activation and local immune modulation, urinary cytokines appear to capture downstream or indirect reflections of tumor-associated inflammation. Plasma cytokines, in contrast, likely represent a composite signal influenced by tumor burden, immune activation, and systemic inflammatory conditions [95].

These compartment-dependent differences underscore the importance of interpreting cytokine data in the context of their biological origin and functional relevance. The existence of cytokine–STAT3 crosstalk across compartments provides a biological rationale for integrative, multi-matrix biomarker strategies in ccRCC [15,88].

### 6.5. Biological and Translational Implications

The cumulative evidence suggests that cytokine–STAT3 interactions may represent an important component of the biological processes linking inflammation, immune modulation, and tumor progression in ccRCC [96,97]. Rather than serving as a passive downstream effector, available evidence supports a role for STAT3 as a potential integrator of cytokine-derived signals within the TME [97].

From a translational perspective, these findings highlight STAT3 activation as a functional readout of cytokine network activity and support its relevance for biomarker development [93]. Understanding cytokine–STAT3 interactions at the tissue level provides essential context for interpreting cytokine profiles detected in urine or plasma and may improve the biological specificity of non-invasive biomarkers.

Although numerous studies have demonstrated associations between altered cytokine profiles and STAT3 activation in ccRCC, most available evidence remains observational. Therefore, causal relationships between individual cytokines and STAT3-dependent biological effects cannot be conclusively established from current data. Future mechanistic studies employing pathway inhibition, genetic manipulation, spatial transcriptomics, or single-cell approaches will be necessary to validate functional cytokine–STAT3 interactions within the ccRCC tumor microenvironment.

## 7. Cytokine Profiles in Tissue, Urine, and Plasma: Evidence from ccRCC Patients

Increasing evidence indicates that cytokine-driven inflammatory processes in ccRCC extend beyond the local TME and can be detected across multiple biological compartments. Comprehensive profiling of cytokines in tumor tissue, plasma, and urine provides a multidimensional view of tumor-associated inflammation and offers important insights into the systemic and organ-specific manifestations of ccRCC. Importantly, analysis across compartments allows for the identification of cytokine patterns that may be particularly suitable for diagnostic and prognostic applications.

### 7.1. Cytokine Alterations in Tumor Tissue

Tumor tissue represents the primary site of cytokine production and signaling in ccRCC. Comparative analyses between malignant and non-malignant renal tissue have demonstrated significant alterations in the expression of multiple cytokines, reflecting both tumor cell–intrinsic changes and microenvironmental remodeling. These alterations encompass cytokines involved in pro-inflammatory signaling, immune regulation, angiogenesis, and tissue remodeling [90,98].

Importantly, tissue-based cytokine profiling reveals that ccRCC is characterized by a complex inflammatory signature rather than uniform upregulation of a single cytokine. Multiple cytokines exhibit significantly altered levels, underscoring the coordinated nature of inflammatory signaling within the TME. Such patterns are consistent with the concept that tumor-associated inflammation in ccRCC is maintained by interconnected cytokine networks rather than isolated signaling events [90,99]. Recent tissue-based cytokine profiling studies in ccRCC have demonstrated increased intratumoral expression of several inflammatory mediators, including IL-6, IL-10, CXCL10, and CCL3, compared with non-malignant renal tissue [89,100].

Assessment of activated STAT3 within tumor tissue provides additional functional context for these observations. Elevated levels of phosphorylated STAT3 have been detected in ccRCC tissue compared with non-malignant controls, supporting the presence of sustained cytokine-driven signaling activity. Moreover, significant correlations between STAT3 activation and the levels of selected cytokines highlight the biological relevance of cytokine–STAT3 crosstalk in situ [101]. These associations support the concept that local cytokine signaling contributes to sustained STAT3-driven transcriptional activity within the TME [89].

Notably, differences in tissue cytokine profiles have also been observed between early-stage and advanced-stage ccRCC. Such stage-dependent variations indicate that inflammatory signaling evolves during disease progression and may contribute to increasing tumor aggressiveness [14,15,102]. The association between specific cytokine patterns, STAT3 activation, and tumor stage further supports the potential utility of tissue-based inflammatory markers for prognostic stratification.

### 7.2. Urinary Cytokine Profiles and Their Clinical Relevance

Urine represents a particularly attractive biological matrix for biomarker discovery in renal malignancies due to its direct contact with the tumor-bearing organ and its accessibility through non-invasive sampling. In patients with ccRCC, urinary cytokine profiling has revealed significant alterations in the levels of multiple cytokines compared with control individuals, suggesting that tumor-associated inflammatory processes are reflected in the urinary compartment [103]. Specifically, several inflammatory mediators, including IL-6, GM-CSF, CXCL10, and homodimer of platelet-derived growth factor B chains have been detected at altered levels in urine samples from patients with ccRCC [84,89].

Importantly, urinary cytokine profiles appear to capture disease-associated information that is not fully mirrored by plasma measurements, underscoring the compartment-specific nature of cytokine signaling and highlighting the potential relevance of urinary cytokines as non-invasive biomarkers in ccRCC. Furthermore, the limited short-term changes in urinary cytokine levels following surgical tumor removal suggest that certain inflammatory signals may persist beyond immediate tumor excision, potentially reflecting residual tissue remodeling or systemic immune responses [89,90,100].

Differences in urinary cytokine profiles have also been observed in relation to tumor stage, with distinct patterns emerging between early and advanced disease. These findings indicate that urinary cytokines may serve as indicators of tumor burden and biological aggressiveness. From a translational perspective, such stage-associated differences enhance the potential relevance of urinary cytokine signatures for risk stratification and disease monitoring [82,94].

Interpretation of urinary cytokine measurements requires careful consideration of several methodological factors. Urinary cytokine concentrations may be influenced by urine dilution, hydration status, and normalization strategy, including adjustment to urinary creatinine concentration or specific gravity. In addition, renal function, hematuria, urinary tract infections, and perioperative inflammatory changes may substantially affect urinary cytokine levels independent of tumor biology. These factors should be considered when comparing results across studies and when evaluating the potential clinical utility of urinary cytokine biomarkers.

### 7.3. Plasma Cytokines as Systemic Inflammatory Indicators

Plasma cytokine levels provide insight into the systemic inflammatory response associated with ccRCC. Alterations in circulating cytokine levels have been reported in patients with ccRCC, although changes observed in plasma tend to be less pronounced than those detected in tumor tissue or urine, likely reflecting the dilution of tumor-derived signals within the systemic circulation [65,104].

Systemic cytokine profiles likely reflect a combination of tumor-associated inflammatory signaling and broader immune activation, which may limit their tissue specificity and sensitivity for early disease detection. Nevertheless, significant changes in the levels of selected cytokines have been detected in the plasma of patients with ccRCC, indicating that local tumor-associated inflammation can exert measurable systemic effects [104]. As a result, plasma cytokines may hold value as complementary biomarkers reflecting overall inflammatory status or treatment response rather than as standalone diagnostic indicators.

The limitations apply to plasma cytokine analyses. Circulating cytokine concentrations may be affected by systemic inflammatory conditions, autoimmune diseases, obesity, concomitant medications, and other comorbidities. Furthermore, plasma cytokine levels may reflect not only local tumor-associated inflammation but also tumor burden, metastatic disease, and generalized immune activation. Consequently, interpretation of plasma cytokine profiles requires careful adjustment for potential confounding factors.

### 7.4. Integrative Analysis Across Biological Compartments

Comparative evaluation of cytokine profiles across tumor tissue, urine, and plasma highlights both concordant and divergent patterns of inflammatory signaling in ccRCC. While some cytokines exhibit parallel alterations across compartments, others display compartment-specific behavior, emphasizing the importance of multi-matrix analyses [90,105].

The integration of tissue-based cytokine expression with urinary and plasma profiles provides a more comprehensive understanding of cytokine–STAT3 signaling dynamics in ccRCC. Correlations between tissue cytokines and their counterparts in urine or plasma support the notion that local inflammatory processes can be partially captured by non-invasive sampling. At the same time, discrepancies between compartments underscore the complexity of cytokine regulation and the need for careful interpretation of biomarker data [89,90,100,105].

Taken together, cytokine profiling across tumor tissue, urine, and plasma suggests that inflammatory signaling in ccRCC is highly compartment-specific [44]. While intratumoral cytokines are closely linked to STAT3 activation and local immune modulation, urinary cytokines may provide a non-invasive readout of tumor-associated inflammatory processes [84,89]. These compartment-dependent patterns underscore the importance of integrative biomarker strategies that account for both local and systemic inflammatory signaling. Differences observed between tissue, urine, and plasma compartments may therefore reflect both biological compartmentalization and methodological variability, emphasizing the need for standardized sampling and analytical procedures. To facilitate interpretation of the available evidence and to distinguish between mechanistic, translational, and biomarker-oriented studies, representative investigations supporting cytokine-mediated inflammatory signaling and biomarker development in ccRCC are summarized in Table 2. In addition, representative cytokine patterns across tumor tissue, urine, and plasma are compared in Table 3 to illustrate the compartment-specific nature of inflammatory signaling in ccRCC.

As summarized in Table 2, current evidence supporting cytokine–STAT3 interactions in ccRCC spans multiple levels of investigation, ranging from translational studies of tumor immune biology to exploratory biomarker analyses in tissue, plasma, urine, and peripheral blood. While these studies collectively support a biologically plausible role of cytokine-mediated inflammatory signaling in ccRCC, most biomarker-related findings remain observational and require independent validation before routine clinical implementation. The compartment-specific nature of these inflammatory signatures across tumor tissue, urine, and plasma is further illustrated in Table 3.

### 7.5. Implications for Biomarker Development

Collectively, cytokine profiling across tissue, urine, and plasma underscores the potential of inflammatory signatures as biomarkers in ccRCC [65,88]. Tissue-based analyses offer mechanistic insight and prognostic information, while urine-based cytokine profiles provide a promising avenue for non-invasive assessment of tumor-associated inflammation. Plasma cytokines may serve as complementary indicators of systemic immune activation.

Importantly, the biological relevance of cytokine alterations is enhanced when interpreted in conjunction with downstream signaling activity, such as STAT3 activation [14,15,106]. Integrative biomarker strategies that combine cytokine measurements with functional signaling readouts may therefore offer improved specificity and clinical utility.

## 8. Diagnostic and Prognostic Implications of Cytokine–STAT3 Signaling in ccRCC

The identification of reliable biomarkers for early detection, risk stratification, and disease monitoring remains a major unmet need in the management of ccRCC [18]. Despite advances in imaging and molecular characterization, no clinically validated biomarker has yet been incorporated into routine practice for the early diagnosis of ccRCC or for precise prognostic assessment. In this context, cytokine–STAT3 signaling represents a biologically plausible and clinically relevant axis that warrants careful evaluation as a potential source of clinical and prognostic information [65,103].

### 8.1. Diagnostic Considerations

From a clinical perspective, cytokines offer several theoretical advantages as biomarkers. They are soluble, quantifiable molecules that reflect dynamic biological processes within the TME and can be detected in multiple biological matrices. However, their clinical utility is constrained by biological variability, pleiotropic effects, and potential confounding by non-malignant inflammatory conditions. These limitations underscore the importance of cautious interpretation and validation in well-designed clinical studies [65,107].

In ccRCC, tissue-based cytokine alterations provide strong evidence of tumor-associated inflammatory remodeling but are inherently limited by the invasive nature of tissue sampling. While such analyses are valuable for mechanistic insight and prognostic evaluation, their applicability for early diagnosis is restricted [108,109]. In contrast, non-invasive matrices such as urine and plasma are more attractive for diagnostic purposes, particularly in populations at increased risk of RCC [110].

Urinary cytokine profiling is of particular interest in renal malignancies due to the anatomical and functional relationship between the kidney and the urinary compartment. Altered urinary cytokine patterns observed in ccRCC patients suggest that tumor-associated inflammatory processes may be captured non-invasively [89,90]. Importantly, urinary cytokine profiles appear to provide information that is not fully recapitulated by plasma measurements, indicating potential added detection value non-invasively [111]. Nevertheless, further studies are required to determine the sensitivity, specificity, and reproducibility of urinary cytokine signatures in comparison with currently available clinical methods.

### 8.2. Prognostic Implications

Prognostic stratification in ccRCC currently relies primarily on clinicopathological parameters, including tumor stage, histological grade, and established risk scoring systems. While these tools provide useful population-level estimates, they do not fully capture the biological heterogeneity of the disease [88]. Cytokine–STAT3 signaling may offer complementary prognostic information by reflecting the inflammatory and immunological state of the tumor.

Associations between altered cytokine levels, STAT3 activation, and advanced disease stage suggest that inflammatory signaling intensity may correlate with tumor aggressiveness. Such observations raise the possibility that cytokine–STAT3 profiles could aid in identifying patients with biologically more aggressive tumors, even within the same clinicopathological stage. However, given the complexity of cytokine networks and their susceptibility to systemic influences, prognostic application should be approached with caution [112,113].

Urinary cytokines may hold particular promise for longitudinal monitoring, as repeated sampling is feasible without additional patient burden. Changes in urinary cytokine patterns over time could potentially reflect disease progression or response to therapy [110]. At present, these applications remain exploratory, and prospective validation is essential before clinical implementation can be considered.

### 8.3. Integrating Cytokine and STAT3 Readouts

An important consideration in biomarker development is the integration of upstream signaling mediators with downstream functional readouts. Cytokine measurements alone provide limited insight into signaling activity, whereas assessment of STAT3 activation offers a functional perspective on the biological impact of cytokine signaling. Combining cytokine profiles with indicators of STAT3 activation may therefore enhance the interpretability and robustness of biomarker strategies [17,114].

Such integrative approaches may reduce reliance on single-marker measurements and better reflect the complexity of tumor-associated inflammation. However, standardization of assays, definition of clinically meaningful thresholds, and validation across independent cohorts remain critical challenges.

### 8.4. Limitations and Need for Validation

Despite promising biological rationale, several limitations must be acknowledged when considering cytokine–STAT3 signaling as a clinical biomarker axis in ccRCC. Cytokine levels are influenced by numerous factors, including comorbid conditions, renal function, and perioperative changes. STAT3 activation, while biologically informative, requires tissue-based assessment in most current applications, limiting its diagnostic accessibility [109].

Therefore, any proposed clinical use of cytokine or STAT3-based biomarkers must be supported by rigorous validation in large, well-characterized patient cohorts. Multicenter studies and standardized analytical protocols will be essential to determine their true clinical value.

Additional challenges include heterogeneity in cytokine assay platforms, differences in sample processing protocols, variability in normalization approaches, and inconsistent adjustment for clinical confounders across studies. These factors currently limit direct comparison between studies and complicate translation into clinical practice.

## 9. Future Directions

The growing body of evidence highlights cytokine–STAT3 signaling as a central component of the immunobiochemical landscape of ccRCC. Rather than acting as isolated mediators, cytokines form complex and dynamic networks that integrate signals from tumor cells, immune infiltrates, and stromal elements [96]. STAT3 emerges as a key molecular hub within these networks, translating diverse extracellular inflammatory cues into coordinated transcriptional programs that support tumor progression, angiogenesis, and immune modulation [106].

A major strength of the cytokine–STAT3 axis lies in its ability to reflect both local tumor-associated inflammation and its systemic manifestations. Analysis across multiple biological compartments underscores the compartment-specific nature of cytokine signaling in ccRCC [88,89,90]. Tumor tissue provides mechanistic insight into inflammatory remodeling and signaling activity, while urine offers a non-invasive window into disease-associated processes occurring within the tumor-bearing organ. Plasma cytokines, although less specific, may complement these approaches by capturing broader systemic immune responses. Integrative assessment across compartments therefore represents a promising strategy for advancing biomarker research in ccRCC.

Future research efforts should focus on several key areas. First, larger prospective studies are needed to validate cytokine signatures and STAT3 activation patterns in independent patient cohorts and across different disease stages. Emerging technologies, including single-cell cytokine profiling and spatial transcriptomic approaches, may further improve understanding of compartment-specific inflammatory signaling and cellular heterogeneity within the ccRCC microenvironment. Second, longitudinal analyses will be essential to determine whether changes in cytokine profiles, particularly in urine, can reliably reflect disease progression, therapeutic response, or recurrence. Third, improved standardization of analytical methods and harmonization of study designs will be critical for translating experimental findings into clinically meaningful tools.

From a translational perspective, targeting cytokine–STAT3 signaling remains an area of active investigation. While direct inhibition of STAT3 has proven challenging, modulation of upstream cytokine signaling or combination strategies with existing immunotherapies may offer indirect means of disrupting this axis [14]. A deeper understanding of cytokine–STAT3 crosstalk may therefore inform the rational design of future therapeutic approaches aimed at reshaping the TME and overcoming immune resistance.

## 10. Conclusions

In conclusion, cytokine–STAT3 signaling represents a biologically relevant and clinically intriguing axis in ccRCC. Although significant challenges remain before cytokine- or STAT3-based biomarkers can be implemented in routine clinical practice, integrative approaches that combine cytokine profiling with functional signaling readouts hold promise for improving disease characterization. Continued investigation of this complex signaling network may ultimately contribute to more precise diagnostic strategies and more effective, personalized management of ccRCC.

## Figures and Tables

**Table 1:** Functional classification of cytokines and their physiological effects.

Class	Functional Subgroups	Physiological Effects
Transforming Growth Factors (TGFs) [38,39]	TGF-β	Stimulation of fibroblast proliferation and extracellular matrix production; inhibition of matrix metalloproteinase expression
Hematopoietins [40]	G-CSF, GM-CSF, M-CSF	Stimulation of proliferation and differentiation of myeloid progenitor cells
	Erythropoietin	Regulation of erythropoiesis
	Thrombopoietin	Regulation of megakaryocyte proliferation and platelet production
Interleukins (ILs) [39,41,42]	IL-3, IL-7, Flt3 ligand	Stimulation of hematopoiesis
	IL-1, IL-6	Pleiotropic pro-inflammatory effects
	IL-2, IL-4, IL-5, IL-12, IL-13	Regulation of T- and B-lymphocyte cooperation and differentiation
Chemokines [43]	α: CXC	Chemotaxis of granulocytes and lymphocytes; stimulation of angiogenesis and inflammation
	β: CC	Chemotaxis of monocytes
	γ: C, δ: CX3C	Chemotaxis of lymphocytes
Interferons (IFNs) [39,40]	Type I: IFN-α, IFN-β, IFN-ε, IFN-κ, IFN-ω, IFN-δ, IFN-τ	Antiviral immunity; antiproliferative effects
	Type II: IFN-γ	Antitumor activity; immune response to intracellular pathogens
	Type III: IFN-λ1–4	Antiviral immunity
Tumor Necrosis Factors (TNF) [39]	TNF-α (cachectin)	Pro-inflammatory and pyrogenic effects; activation of innate immunity; induction of endothelial adhesion molecules; apoptosis; systemic inflammatory response syndrome
	TNF-β (lymphotoxin-α)	Effects similar to TNF-α

TGF-β—transforming growth factor beta; G-CSF—granulocyte colony-stimulating factor; GM-CSF—granulocyte–macrophage colony-stimulating factor; M-CSF—macrophage colony-stimulating factor; IL-3—interleukin-3; IL-7—interleukin-7; Flt3 ligand—Fms-like tyrosine kinase 3 ligand; IL-1—interleukin-1; IL-6—interleukin-6; IL-2—interleukin-2; IL-4—interleukin-4; IL-5—interleukin-5; IL-12—interleukin-12; IL-13—interleukin-13; CXC—CXC motif chemokines; CC—CC motif chemokines; C—C motif chemokines; CX3C—CX3C motif chemokines; IFN-α—interferon alpha; IFN-β—interferon beta; IFN-ε—interferon epsilon; IFN-κ—interferon kappa; IFN-ω—interferon omega; IFN-δ—interferon delta; IFN-τ—interferon tau; IFN-γ—interferon gamma; IFN-λ1–4—interferon lambda 1–4; TNF-α—tumor necrosis factor alpha; TNF-β—tumor necrosis factor beta.

**Table 2:** Representative studies supporting cytokine–STAT3 signaling and biomarker development in clear cell renal cell carcinoma.

Study Design	Biological Material	Main Objective	Principal Findings	Evidence Level	Major Limitations
Translational immune profiling study [88]	Human ccRCC tumor tissue	Characterization of immune dysfunction across disease stages	Progressive immune dysregulation was associated with advancing ccRCC stage, highlighting the importance of the tumor immune microenvironment	Translational mechanistic evidence	Not specifically focused on cytokine–STAT3 signaling
Exploratory clinical study [89]	Tumor tissue	Characterization of the immunobiochemical profile of ccRCC	Distinct cytokine alterations were identified in ccRCC tissue and were associated with tumor-associated immune responses	Tissue-based clinical association	Preliminary observational study
Clinical translational study [90]	Tumor tissue and plasma	Comparison of local and systemic cytokine profiles	Demonstrated compartment-specific cytokine signatures and differences between tumor-derived and circulating inflammatory mediators	Translational clinical evidence	Predominantly correlational findings; external validation required
Clinical translational study [84]	Urine and plasma	Evaluation of immune and cytokine profiles in ccRCC	Distinct immune alterations were identified in urine and plasma, supporting compartment-specific inflammatory responses	Exploratory biomarker evidence	Clinical utility requires further validation
Clinical biomarker study [104]	Peripheral blood	Identification of circulating immune biomarkers associated with immunotherapy response	Several circulating immune biomarkers correlated with treatment response in metastatic RCC patients receiving immunotherapy	Clinical biomarker evidence	Prospective external validation remains necessary

**Table 3:** Comparative Overview of Cytokine Profiles in Tumor Tissue, Urine, and Plasma in ccRCC.

Cytokine	Tumor Tissue	Urine	Plasma	Potential Biological Relevance
**IL-6**	↑	↑	↑	STAT3 activation, tumor progression, systemic inflammation
**IL-10**	↑	Variable	Variable	Immunosuppression and immune regulation
**CXCL10**	↑	↑	Variable	Immune-cell recruitment and inflammatory signaling
**G-CSF**	↑	Variable	Variable	Myeloid-cell recruitment and immune modulation
**CCL3**	↑	Not consistently reported	Variable	Tumor-associated inflammation and immune-cell trafficking

## Data Availability

Data sharing is not applicable to this article as no new data were created or analyzed in this study.

## Abbreviations

The following abbreviations are used in this manuscript:

BCL-2	B-cell lymphoma 2 (antiapoptotic protein)
BCL-XL	B-cell lymphoma-extra-large (antiapoptotic protein)
CC	CC chemokine family
CCL	CC motif chemokine ligand
CD8^+^	Cluster of differentiation 8 positive T cells (cytotoxic T lymphocytes)
c-MYC	MYC proto-oncogene (transcription factor regulating proliferation)
CXC	CXC chemokine family
CX3C	CX3C chemokine family
CXCL	CXC motif chemokine ligand
DNA	Deoxyribonucleic acid
ECM	Extracellular matrix (structural component of the TME)
ELR	Glutamic acid–leucine–arginine motif (pro-angiogenic sequence in chemokines)
EMT	Epithelial–mesenchymal transition (process promoting invasion and metastasis)
G-CSF	Granulocyte colony-stimulating factor (hematopoietic growth factor)
GM-CSF	Granulocyte–macrophage colony-stimulating factor (immune cell regulator)
GPCRs	G protein-coupled receptors (cell surface signaling receptors)
HIF	Hypoxia-inducible factor (transcription factor activated by hypoxia)
HIF-1α	Hypoxia-inducible factor 1 alpha (key regulator of hypoxic response)
HIFs	Hypoxia-inducible factors (family of hypoxia-responsive transcription factors)
IFN	Interferon (cytokine involved in immune responses)
IFN-γ	Interferon gamma (pro-inflammatory cytokine)
IFNs	Interferons (family of immune-regulating cytokines)
IL	Interleukin (cytokine family involved in immune regulation)
IL-6	Interleukin 6 (pro-inflammatory cytokine linked to STAT3 activation)
IL-8	Interleukin 8 (pro-angiogenic chemokine)
ILs	Interleukins (group of cytokines with diverse immune functions)
JAK	Janus kinase (cytoplasmic tyrosine kinase)
JAKs	Janus kinases (family of signaling kinases)
MCL-1	Myeloid cell leukemia 1 (antiapoptotic protein)
M-CSF	Macrophage colony-stimulating factor (regulator of macrophage differentiation)
MDSC	Myeloid-derived suppressor cell (immunosuppressive cell population)
MMP	Matrix metalloproteinase (enzyme degrading extracellular matrix)
MMPs	Matrix metalloproteinases (enzymes involved in tissue remodeling and invasion)
NK	Natural killer cells (innate immune cytotoxic cells)
PDGF-BB	Platelet-derived growth factor BB (growth factor promoting proliferation and angiogenesis)
PIAS	Protein inhibitor of activated STAT (negative regulator of STAT signaling)
p-STAT3	Phosphorylated STAT3 (active form of STAT3)
RCC	Renal cell carcinoma
SIRS	Systemic inflammatory response syndrome
SOCS	Suppressor of cytokine signaling (negative feedback regulator of cytokine pathways)
STAT3	Signal transducer and activator of transcription 3 (transcription factor mediating cytokine signaling)
TAM	Tumor-associated macrophage (pro-tumor immune cell)
TAMs	Tumor-associated macrophages (immunosuppressive macrophage population)
TGF	Transforming growth factor (cytokine regulating cell growth and immune responses)
TGF-β	Transforming growth factor beta (immunosuppressive cytokine)
TIL	Tumor-infiltrating lymphocyte (immune cells within the tumor)
TME	Tumor microenvironment (complex cellular environment of the tumor)
TNF	Tumor necrosis factor (pro-inflammatory cytokine)
TNF-α	Tumor necrosis factor alpha (key mediator of inflammation)
TNF-β	Tumor necrosis factor beta (lymphotoxin involved in immune regulation)
TK	Tyrosine kinase (enzyme mediating signal transduction)
Tregs	Regulatory T cells (immunosuppressive T-cell subset)
VEGF	Vascular endothelial growth factor (key mediator of angiogenesis)
VHL	Von Hippel–Lindau tumor suppressor gene
YAP/TAZ	YAP (Yes-associated protein) and TAZ (transcriptional coactivator with PDZ-binding motif)
ZEB1	Zinc finger E-box-binding homeobox 1 (transcription factor promoting EMT)
